# Correlative Analysis of miRNA Expression and Onco*type* Dx Recurrence Score in Estrogen Receptor Positive Breast Carcinomas

**DOI:** 10.1371/journal.pone.0145346

**Published:** 2015-12-30

**Authors:** Rajyasree Emmadi, Emanuele Canestrari, Zarema H. Arbieva, Wenbo Mu, Yang Dai, Jonna Frasor, Elizabeth Wiley

**Affiliations:** 1 Department of Pathology, University of Illinois at Chicago, Chicago, Illinois, United States of America; 2 Center for Pharmaceutical Biotechnology, University of Illinois at Chicago, Chicago, Illinois, United States of America; 3 Core Genomics Facility, University of Illinois at Chicago, Chicago, Illinois, United States of America; 4 Department of Bioengineering, University of Illinois at Chicago, Chicago, Illinois, United States of America; 5 Department of Physiology and Biophysics, University of Illinois at Chicago, Chicago, Illinois, United States of America; West Virginia University, UNITED STATES

## Abstract

Altered expression of miRNAs has been observed in many types of cancer, including breast cancer, and shown to contribute to cancer growth, aggressiveness, and response to therapies. In this pilot study, we investigated the possible correlation of miRNAs with risk of recurrence of estrogen receptor positive, lymph node-negative mammary carcinomas as determined by the Onco*type* DX^®^ Breast Cancer assay. To accomplish this, we extracted RNA from a collection of breast carcinomas that had previously been analyzed by Onco*type* DX^®^. Multiple Let-7 family members were negatively correlated with the recurrence score (RS), which is consistent with their tumor suppressor properties. Additional miRNAs were found to positively correlate with RS, including miR-377-5p, miR-633b, miR-548t and miR-3648. Pathway analysis of putative and validated targets suggests that these miRNAs may have a diverse range of functions that may contribute to tumor recurrence. Taken together, these findings provide evidence that a miRNA expression signature can be developed to aid existing methods to determine the risk of recurrence for women with estrogen receptor positive breast cancers treated with endocrine therapy.

## Introduction

Invasive breast tumors are biologically diverse with different rates of progression, treatment response, and outcomes. The current standard of care requires all newly diagnosed cases of invasive breast cancer to be routinely tested by immunohistochemistry (IHC) for the expression of estrogen receptor alpha (ER), progesterone receptor (PR) and the growth factor receptor *HER2/neu* (*ERBB2*). ER-positive tumors are treated with endocrine therapy, such as tamoxifen or aromatase inhibitors. However, about one third to one half of patients treated with tamoxifen or other endocrine therapy will develop recurrence, in part due to development of acquired endocrine resistance. The differential responsiveness of ER-positive breast cancers to endocrine therapy is explained by the heterogeneity of these tumors, which has been clearly demonstrated by genome-wide expression profiling studies. The two sub-classifications of ER-positive tumors, representing biologically distinct tumor types, luminal A and luminal B, have been identified, with the luminal A tumors having a good response to endocrine therapy and luminal B tumors responding poorly [1, 2].

Based on gene expression studies, the Onco*type* DX^®^ assay was developed through the NSABP clinical trials [3]. Since 2007, the Association for Clinical Oncology (ASCO) Guidelines have used Onco*type* DX^®^ testing for treatment stratification of ER-positive, lymph node negative breast carcinomas [4]. Onco*type* DX^®^ is a reverse transcription polymerase chain reaction (RT-PCR) based assay that is performed on RNA isolated from formalin fixed paraffin embedded (FFPE) tumor tissue blocks. Based on the expression of 21 genes in the tumor, a Recurrence Score (RS) is issued. The 16 cancer genes included in the assay are associated with proliferation (*Ki67*, *STK15*, *Survivin*, *Cyclin B1*, *MYBL2*), estrogen action (*ER*, *PR*, *Bcl-2*, *SCUBE2*), *HER2/cERBB2* action (*GRB7*, *HER2*), invasion (*Stromelysin 3*, *Cathepsin L2*), or other functions (*GSTM1*, *CD68 and BAG1*). Five reference genes (*beta-actin*, *GADPH*, *RPLPO*, *GUS* and *TFRC*) are also included in the assay. The recurrence score is represented by a number between 0 and 100, which correlates to a specific likelihood of breast cancer recurrence within 10 years of initial diagnosis. The scores are grouped into 3 risk categories (low: RS ≤17, intermediate: RS = 18–30, and high: RS>31). Oncologists use the RS to tailor the use of chemotherapies based on the finding that women with high RS benefit from chemotherapy whereas women with low RS do not [5]. Hence, women at low risk for recurrence may avoid the toxicity of chemotherapy and be successfully managed with hormonal therapy alone. Patients with high RS, on the other hand, are at an increased risk of recurrence and may receive one or both types of therapy. Little is known about what distinguishes the intermediate from the high RS groups so they generally receive similar therapies. In fact, in 2006, the National Cancer Institute (NCI) sponsored a prospective randomized clinical trial, the TAILORx trial, involving hormone-receptor-positive, *HER2*-negative, axillary node-negative breast cancers less than 5.0 cm in size, assigned to one of three treatment arms based on their On*cotype*DX RS results. The results of this trial have recently been released, showing that patients with tumors that had favorable gene-expression profiles (RS of 0–10) had very low rates of recurrence at 5 years with endocrine therapy alone. Of note, however, is that new cut-offs were used to designate the three subgroups of RS compared to the original On*cotype*DX assay. The new RS ranges were 0–10 (low risk), 11–25 (intermediate risk) and ≥26 (high risk), in contrast to prior ranges of <18, 18–30 and ≥31, respectively. The overall sample size of 10,253 women was driven by the need to include a sufficient number of patients in the ‘new’ intermediate risk range of RS of 11–25.

In addition to gene expression signatures, recent studies have revealed associations between miRNAs and tumor expression of *ER*, *PR* and *HER2*, as well as breast carcinoma subtypes and tumor grades [6, 7]. In addition to these correlative studies, specific miRNAs have been shown to play key roles in hormone-dependent breast cancer biology. For example, down-regulation of miR-451 can promote breast cancer cell survival and endocrine resistance [8], a miRNA196a2-TP63 circuit controls breast cancer proliferation and invasiveness properties [9], loss of miR-200c promotes breast cancer cell migration and invasion [10], and down-regulation of miR-29 contributes to progestin-induced stem cell expansion [11]. Let-7 family members have been found to inhibit breast cancer stem cell renewal [12] and have been implicated in tamoxifen response through modulation of ER levels [13]. Furthermore, miRNAs that are differentially expressed in tamoxifen-sensitive vs. resistant cell lines and tumors have been identified [14, 15]. These findings suggest that miRNAs may function as biomarkers of tumor response to therapy and/or risk of recurrence, as well as important drivers of these phenotypically different tumors. In this study we conducted miRNA profiling in a collection of breast tumors with previous testing for recurrence risk by the Onco*type* DX^®^ assay in an attempt to identify additional miRNAs biomarkers of breast tumor recurrence and poor outcome in patients with ER-positive breast cancers. This is a pilot study with a small sample size. However, cases were selected to span the range of the Onco*type* DX^®^ Recurrence Scores. We acknowledge the limitations of the study including retrospective analysis and a small sample size.

## Materials and Methods

### Patient Cohort and Tumor Characteristics

This research was a retrospective, non-interventional analysis, conducted following approval from the Institutional Review Boards (IRBs) of the University of Illinois Cancer Center and Provena Saint Joseph Medical Center in Illinois. The research was judged to qualify for waiver of informed consent based on the provisions under HHS regulations at 45 CFR 46.116(d). The research did not involve any risk to patients, data was anonymized and no patient identifiers were included. Formalin-fixed, paraffin-embedded (FFPE) tumor tissues from twenty-three cases of early stage breast carcinomas representing low, intermediate and high Onco*type* DX Recurrence Score were obtained. Patient samples and clinical data were collected and processed in compliance with protocols approved by the University of Illinois Cancer Center and Provena Saint Joseph Medical Center Institutional Review Boards. Tumor tissue was macro-dissected by comparison to an adjacent H&E stained section to ensure that tissue used for miRNA analysis contained >70% tumor, in accordance with samples sent for Onco*type* DX^®^ testing. Clinical data collected on each patient included information on ER, PR, p53, Ki67 and Her2 status for correlation with study results (Table 1).

**Table 1:** Patient and tumor characteristics.

ID	RS	Group	Recurrence Risk (%)	ER	PR	p53	Ki67	Her2	Age	Race
S2	3	Low	4	81	52	13	0	1.7	54	White
S53	6	Low	5	30	75	0	6	0	46	White
S70	7	Low	6	97	50	9	16	1.1	45	AA
S46	8	Low	6	20	9	0	2	0.2	58	White
S30	9	Low	6	93	63	0	8	0	62	White
S21	11	Low	8	50	48	0	2	0	73	White
S11	12	Low	8	66	57	0	7	0.5	60	White
S20	12	Low	8	56	18	1	4	0.6	52	White
S7	14	Low	9	29	19	10	23	0.9	57	White
S23	14	Low	9	87	85	1	8	1	54	White
S63	14	Low	9	72	78	0	7	1.4	52	White
S33	16	Low	10	90	68	0	13	0.2	65	White
S26	26	Int	17	98	97	13	53	1	46	White
S29	26	Int	17	1	2	7	3	1.2	70	White
S8	27	Int	17	88	42	16	3	1.12	70	White
S16	29	Int	20	83	71	36	17	0	75	White
S58	31	High	21	37	42	0	9	0.9	50	White
S25	32	High	22	29	86	5	14	1.9	53	White
S12	38	High	26	95	91	14	42	0	47	Asian
S49	39	High	27	58	62	2	21	1.4	55	White
S64	40	High	27	100	37	2	63	1.4	59	White
S6	47	High	32	29	52	0	33	0.3	43	White
S32	56	High	34	23	2	1	31	1.4	48	White

### miRNA Profiling

RNA was extracted from each sample using the FFPE RNA Purification Kit, according to the manufacturer’s instructions (Norgen Biotek Corp., Thorold, ON, Canada). The quality of the total RNA was verified by Agilent 2100 Bioanalyzer profile. Total RNA from each sample was labeled with Hy3 and a reference sample, consisting of equal amounts of total RNA combined from every individual sample, was labeled with Hy5 fluorescent label, using the miRCURY LNAmicroRNA Hi-Power Labeling Kit (Exiqon, Denmark) following the procedure described by the manufacturer. The Hy3-labeled samples and a Hy5-labeled reference sample were mixed pair-wise and hybridized to the miRCURY LNA microRNA Array 6th gen (Exiqon, Denmark), which contains capture probes targeting all microRNAs for human, mouse or rat registered in the miRBASE 16.0. The hybridization was performed using a Tecan HS4800 hybridization station (Tecan, Austria). After hybridization the microarray slides were scanned and stored in an ozone free environment (ozone level below 2.0 ppb) in order to prevent potential bleaching of the fluorescent dyes. The miRCURY LNA microRNA Array slides were scanned using the Agilent G2565BA Microarray Scanner System (Agilent Technologies, Inc., USA) and the image analysis was carried out using ImaGene 9 software (Exiqon, Denmark). The quantified signals were background corrected (Normexp with offset value 10) [16] and normalized using the global Lowess regression algorithm.

### Data Analysis

**Fig 1:**
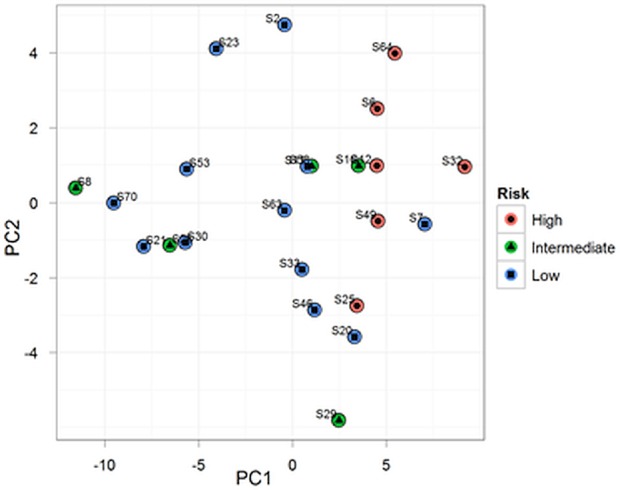
Principal components analysis. Principal components analysis performed on all samples using the top 50 miRNAs showing the highest standard deviation across all samples.

**Fig 2:**
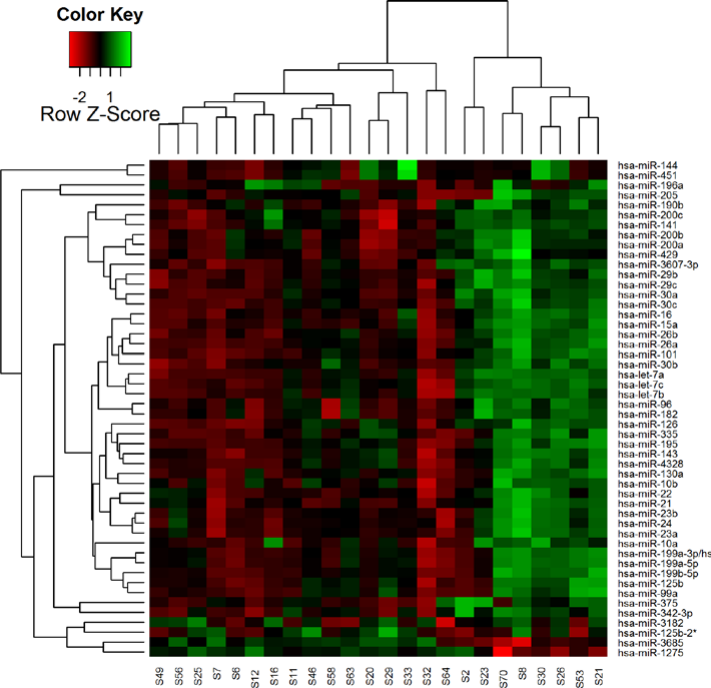
Heat map of unsupervised hierarchical clustering. Hierarchical clustering performed on all samples using the top 50 miRNAs showing the highest standard deviation across all samples.

Principal Components Analysis (PCA) was performed using the top 50 microRNAs that had the largest variation across all samples (Fig 1). A heatmap diagram was generated based on the two-way clustering method with the complete-linkage and Euclidean distance measurement (Fig 2). The intermediate RS group was included in the high RS group since the number of samples in the former was small. Student’s t-test was performed for differential expression analysis between low RS and high RS groups. Pearson correlation coefficient was used for correlation tests between the expression value of individual microRNAs and RS. All p-values were adjusted based on the Benjamini-Hochberg procedure. The threshold for the adjusted p-value <0.05 was used to determine the significant correlation (Table 2). The analysis was carried out using packages in R.

**Table 2:** Differential expression of miRNAs.

	High vs. Low (T-test)		Low vs. Int vs. High (ANOVA)	Correlation Analysis	
miRNA Annotation	Log FC	Adj. P. Val	Adj. P. Val	Correlation Coefficient	Adj. P.Val
hsa-let-7c	-1.506	5.04E-03	0.115	-0.7503	9.22E-03
hsa-let-7b	-1.716	7.35E-03	0.115	-0.7278	1.36E-02
hsa-let-7a	-1.348	2.23E-02	0.115	-0.6774	2.71E-02
hsa-miR-126	-1.108	2.64E-02			
hsa-miR-193a-5p	-0.976	2.95E-02			
hsa-let-7d	-0.933	3.68E-02			
hsa-miR-126*	-0.819	3.68E-02			
hsa-miR-26b	-1.192	3.68E-02			
hsa-miR-30c	-1.167	3.68E-02			
hsa-miR-3648	0.651	3.68E-02		0.7085	1.91E-02
hsa-miR-492	0.539	3.83E-02			
hsa-miR-361-5p	0.340	3.93E-02			
hsa-miR-4328	-1.014	3.93E-02			
hsa-miR-663b	0.631	3.93E-02		0.6837	2.65E-02
hsa-miR-1914	0.332	3.98E-02			
hsa-miR-195	-1.261	3.98E-02			
hsa-miR-663	0.416	3.98E-02			
hsa-miR-29c	-1.314	4.01E-02			
hsa-miR-377-5p	0.235	4.60E-02	4.23E-02	0.7886	3.87E-03
hsa-miR-143	-1.144	4.64E-02			
hsa-miR-26a	-1.032	4.64E-02			
hsa-miR-1297	-0.795	4.80E-02			
hsa-let-7f	-0.962	4.98E-02			
hsa-let-7f-1*	0.253	4.98E-02			
hsa-miR-4301	-0.765	4.98E-02			
hsa-miR-548t				0.6886	2.65E-02

### Predicted and Validated miRNA Targets

**Fig 3:**
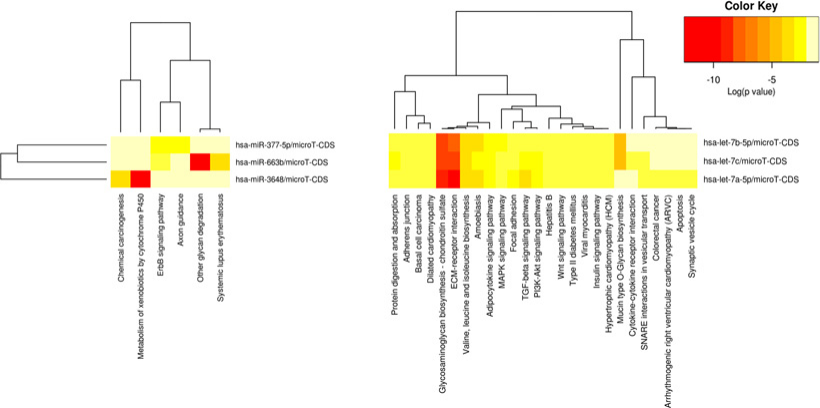
Pathway analysis for putative miRNA target genes. Pathways showing significant enrichment in putative target genes for miRNAs positively correlated with recurrence score (left) or negatively correlated with recurrence score (right) were clustered.

**Fig 4:**
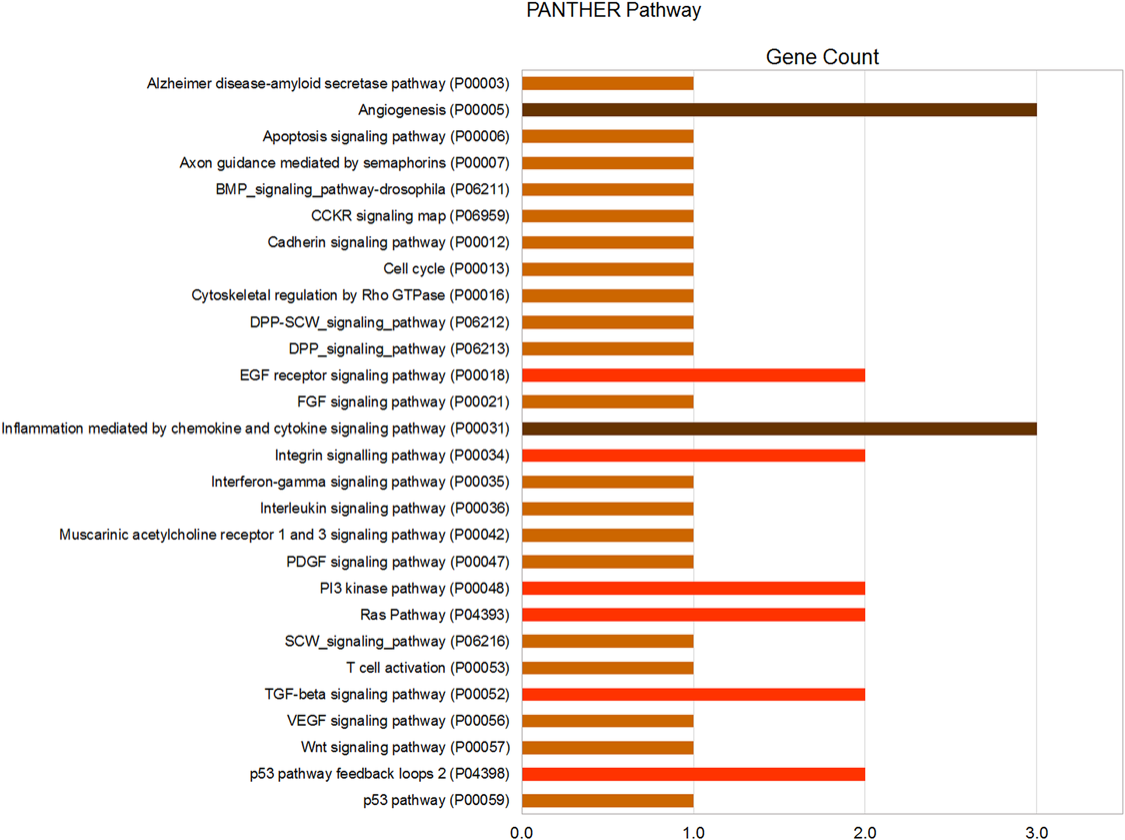
Pathway analysis for validated miRNA target genes. Pathways showing enrichment in validated targets genes for miR-377-5p and miR-663b.

The miRNA story is an evolving one and the currently available databases are still incomplete. In order to maximize the information for correlative analysis, we looked at multiple miRNA databases. Predicted miRNA targets were obtained using DIANA-microT-CDS [17, 18]. Validated miRNA targets were obtained using miRWalk with a default p-value of 0.05 [19]. The findings of the predicted miRNA targets and pathways are presented in Fig 3 and validated targets and pathways are presented in Table 3 and Fig 4.

**Table 3:** Validated gene targets for miR-377-5p and miR-663b using miRWalk.

**miR-377-5p**		
**Gene Name**	**Entrez ID**	**Ensmbl ID**
*LAMA1*	284217	ENSG00000101680
*CHRD*	8646	ENSG00000090539
*MITF*	4286	ENSG00000187098
*NOG*	9241	ENSG00000183691
*CYP7A1*	1581	ENSG00000167910
*CDKN1A*	1026	ENSG00000124762
*IFNG*	3458	ENSG00000111537
*PAK1*	5058	ENSG00000149269
*IL1B*	3553	ENSG00000125538
*PPM1A*	5494	ENSG00000100614
*NOS2A*	4843	ENSG00000007171
*PKN1*	5585	ENSG00000123143
*TNF*	7124	ENSG00000232810
*SOD1*	6647	ENSG00000142168
*HMOX1*	3162	ENSG00000100292
*SOD2*	6648	ENSG00000112096
*DICER1*	23405	ENSG00000100697
*COMP*	1311	ENSG00000105664
*ERBB2*	2064	ENSG00000141736
*GHRHR*	2692	ENSG00000106128
**miR-663b**		
**Gene Name**	**Entrez ID**	**Ensmbl ID**
*EPHB2*	2048	ENSG00000133216
*LATS2*	26524	ENSG00000150457
*KRAS*	3845	ENSG00000133703
*CCND2*	894	ENSG00000118971
*TXNIP*	10628	ENSG00000117289

### Pathway Analysis

#### Predicted targets

miRNA targets were predicted based on DIANA-microT-CDS. The combined effect of groups of miRNAs on pathways was determined based on the union of target genes for enrichment analysis using DIANA miRPath v.2.0 (Vlachos). Heatmaps were generated using the log-transformed enrichment p-values as features for each miRNA. [20].

#### Validated targets

Validated miRNA targets were available only for miR-377-5p (miR-377*) and miR-663b using miRWalk. No gene targets were elucidated for miR-3648 and miR-748t. The gene targets for miR-377-5p and miR-663b were combined and pathway enrichment analysis was performed using the Panther classification system v9.0 and Panther pathway, enriched with a default p-value of 0.05 [21, 22].

## Results

Genome wide profiling of miRNA expression in 23 human breast tumors was carried out using a locked nucleic acid (LNA) based microRNA array that contained ~2800 capture probes complementary to mature microRNAs based on miRBase v16 (Exiqon). All tumors were ER-positive, lymph node-negative, invasive mammary carcinomas. Clinical and laboratory data collected for each patient are shown in Table 1 and include the Recurrence Score (RS) based on the Onco*type* DX^®^ assay and the estimated risk of recurrence (R-Rate). The tumors profiled included 12 low, 4 intermediate and 7 high RS scores.

Principal components analysis (PCA), which was conducted for the top 50 miRNAs with the largest variation in expression across all samples, revealed that the tumors with high or low RS largely clustered independently of each other but that tumors with intermediate RS were more widely distributed (Fig 1). Of the samples with low RS, one (#S7) was more closely related to the high RS samples than other tumors with low RS. Two-way hierarchical clustering by miRNA expression levels confirmed the relationship between samples and recurrence score groups (Fig 2).

**Fig 5:**
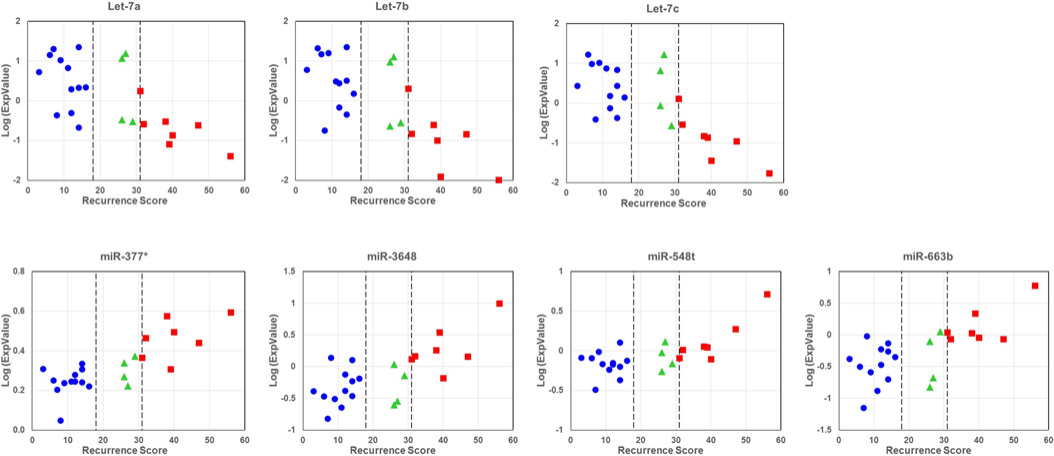
Correlation analysis between miRNA expression level and recurrence score. The relationship between miRNA expression and recurrence score is shown for seven miRNAs with a statistically significant correlation.

To identify specific miRNAs that are associated with RS, two types of analyses were performed. First, differential expression of miRNAs between the low and high RS groups was carried out using Student’s T test followed by Benjamini Hochberg adjustment for multiple tests. Twenty-five miRNAs were found to be statistically different (P<0.05) between the two groups (Table 2). However, when all samples were analyzed as three groups (low, intermediate, and high) using ANOVA and Benjamini Hochberg adjustment, only three of miRNAs remained as significantly different among the groups. In the second approach, correlation analysis between miRNA expression levels and the actual RS was conducted. Expression levels for seven miRNAs were significantly correlated with RS (Fig 5), six of which overlapped with other analyses as indicated in Table 2.

miRNAs showing negative correlation with RS comprise several members of the Let-7 family, which have been found to inhibit breast cancer stem cell renewal [12] and have been implicated in Tamoxifen response through modulation of ER levels [13]. However, little is known about the other miRNAs (miR-377-5p, miR-663b, miR-548T and miR-3648) that we found to be higher in breast tumors with high RS.

To determine pathways that may be controlled by these miRNAs, DIANA miRPath was first used to identify putative targets (see S1 Data for complete results). The union of the putative targets from Let-7a, 7b and 7c (all negatively correlated with RS) and the union of putative targets from miR-663b, miR-377-5p and miR-3648 (all positively correlated with RS) were separately used for pathway enrichment analysis. The miRNAs were then clustered based on the log-transformed enrichment p-values and visualized in heatmaps. As shown in Fig 3, target genes for miRNAs positively correlated with RS are fairly divergent with chemical carcinogenesis and xenobiotic metabolism associated miR-3648, and glycan degradation and systemic lupus erythematosus with miR-633b. *ErbB* signaling, on the other hand, was enriched for both miR-377-5p and miR-633b. In contrast, pathways well characterized in breast cancer progression (*PI3K/AKT*, *MAPK*, cytokines, and *WNT*), are highly enriched for all three Let-7 family members showing positive correlation with RS score.

Validated gene targets obtained for miR-377-5p and miR-663b using miRWalk (Table 3) were then used for pathway enrichment analysis using PANTHER 9.0 and showed association with multiple pathways, in particular those involving angiogenesis, *EGF* receptor signaling, inflammation mediated by chemokines and cytokines, integrin signaling, *PI3 kinase*, *TGF-beta* signaling and *Ras* and p53 pathway feedback loops (Fig 4).

## Discussion

Several publications have looked at the miRNA expression profiles in breast carcinoma cell lines and tumor tissues. Most of the studies have varied representation of the different subtypes of breast carcinoma (luminal, basal-like, Her2-enriched) and with or without lymph node involvement. As a result, mixed (non subtype-specific) miRNA signatures emerge and have been reported in ranges of 1–21 miRNAs [23–27]. Commonly reported differential expressions include upregulation of miR-21, miR-155, miR-206, miR-122a and miR-210 and downregulation of let-7, miR-10b, miR-125a, miR-125b and miR-145. Putative or validated target gene and pathway analyses demonstrate involvement of oncogenes and tumor suppressors, such as *ErbB2*, *Akt*, *NF-κB*, *Myc*, *p53*, *Rb* and *PTEN* with consequent proliferation and cell survival effects as well as inhibition of apoptosis. While cell cycle and proliferation signals would be expected to correlate with outcomes, other signatures described are associated with hypoxia, cytokines, stroma and chromosomal instability. In essence it appears that different reported signatures may represent different aspects of ultimate multifactorial mechanisms. Several of the miRNAs associated with RS in ER+ breast tumors have been described to play a role in cancer progression. For example, Let-7 family members, which in this study were found to be down-regulated in tumors with high recurrence score, have been found to inhibit breast cancer stem cell renewal [12] and have been implicated in tamoxifen response through modulation of ER levels [13]. On the other hand, little is known about the other miRNAs that we find to be higher in breast tumors with high RS. The inferences we have made by correlating the results of our analyses and literature are presented.

1. **miRNA profiling captures basic biological relationship between high vs low risk of recurrence**. More refined risk prediction is needed—both Onco*type* DX^®^ and the MammaPrint^®^ assays have a discordance rate between the assay prediction and clinical-pathologic risk category of approximately 30% [28].
2. **The intermediate group does not represent a unique group biologically but appears to be distributed along a continuum of high to low**. This is similar to findings reported previously by Ivshina et al, who found 6 markers that could effectively separate grade 1 and grade 3 tumors and could also separate grade 2 tumors into two highly discriminate classes [29].
3. **Suppression of the Let-7 family is associated with increased RS score**. Of particular interest is the reduced expression of Let-7 family members in cases with high RS. Six family members were found to be significantly associated with low risk of recurrence using at least one statistical analysis. The hsa-let-7 family is well established as a tumor suppressor with interactions with cell cycle proliferative genes including Aurora Kinase A (*AURKA*), which it downregulates [30]. A recent meta-analysis shows a significant correlation between increased *AURKA* expression and decreased metastasis-free survival in ER-positive breast cancers [31]. The hsa-let-7 family involvement in breast carcinoma is well documented and serves to confirm the validity of our findings.
4. **Novel miRNAs involved in breast carcinoma**. The three miRNAs (hsa-miR-377-5p, hsa-miR-663b and hsa-miR-3648) that are positively correlated with high Onco*type* DX^®^ RS scores have not been previously detailed in association with breast carcinoma outcomes. Very little specific data exists regarding the interactions and roles of these miRNAs with regard to ER-positive breast carcinoma.

Information available via the tools listed suggest that multiple pathways are involved by miR-377-5p and miR-663b, affecting tumor suppressor functions (*LATS2*, *TXNIP*, *wtKRAS*, *CDKN1A*, *PPM1A*), cytoskeleton regulation (*PAK1*, *PKN1*) and a proinflammatory and reactive oxygen species adaptive response (*TNF*, *IFNG*, *IL1B*, *HMOX1*, *SOD1* and *SOD2*). We infer that multiple factors are involved in separating those tumors that have a high RS score including specific proliferation advantages as well as suppression of tumor response mechanisms.

While these genes and pathways may represent possible therapeutic targets for tumor suppression, these miRNAs also are of particular interest as they may provide novel approaches for specific, targeted, less toxic systemic treatment protocols in the future. Progress in microRNA-directed therapeutic approaches offers hope that such strategies might prove useful in reversing endocrine resistance and reducing breast cancer recurrence.

## Supporting Information

S1 Data (XLSX)
